# Acute Chest Syndrome Masquerading As Sepsis: A Rare Presentation of Sickle Cell Anemia Complication

**DOI:** 10.7759/cureus.30208

**Published:** 2022-10-12

**Authors:** Bharathi Mohan, Kavya N P, Arnab Choudhury, Mukesh Bairwa

**Affiliations:** 1 Department of Internal Medicine, All India Institute of Medical Sciences, Dehradun, IND; 2 Department of Internal Medicine, All India Institute of Medical Sciences, Rishikesh, IND

**Keywords:** anemia, sepsis, sickle cell, acute chest syndrome, jaundice

## Abstract

Sickle cell disease in adults leads to various complications by hemolytic anemia and vaso-occlusion. Acute chest syndrome (ACS) is a severe life-threatening complication that can lead to multiple organ dysfunction and rapidly progressing respiratory failure. Early recognition and timely management are vital and lifesaving. We present a case of a 38-year-old female with a past history of multiple blood transfusions, who presented with features suggestive of sepsis with multiple organ dysfunction and obstructive jaundice. The patient showed minimal response to empirical antibiotics. However, no source of infection or features of biliary obstruction was found. The possibility of a hematological disorder was suspected in the background of multiple blood transfusions, and she was eventually diagnosed to have acute chest syndrome. She improved with transfusion, drug therapy, and adequate pain control.

## Introduction

Acute chest syndrome (ACS) is a potentially fatal compilation of sickle cell disease (SCD) which is defined as a new pulmonary infiltrate on chest X-ray accompanied by fever and/ or new respiratory symptoms [1]. ACS is one of the important causes of death and the second most common cause of hospitalization in patients with sickle cell anemia. Data from the Cooperative Study of Sickle Cell Disease (CSSCD) suggest that approximately 50 percent of individuals with SCD will have an episode of ACS. The presentation of ACS can range from mild chest pain to multiple organ dysfunction involving the lung, liver, and/or kidney [2]. We present a case of acute chest syndrome masquerading as sepsis with multiple organ dysfunction.

## Case presentation

A 38-year-old female without any addictions presented to the emergency room with a history of high-grade intermittent undocumented fever for one week. Following this, she developed insidious onset progressive jaundice with high-colored urine, pruritus, and left upper abdominal discomfort. She also developed acute onset gradually progressive shortness of breath without orthopnea or paroxysmal nocturnal dyspnea for five days. There was a history of altered sleep-wake cycle for two days without a history of melena or hematemesis. Past history significant for recurrent red blood cell transfusions (one to two packed red blood cells (PRBC)/ month) since childhood; however, she was not diagnosed with any hematological disease. She had pallor, icterus, and yellow discoloration all over her skin and tachypnoea with an oxygen saturation of 88% on room air. Hepatomegaly was present with a liver span of 20 cm, and the spleen was palpable 10 cm below the left costal margin. Respiratory, cardiovascular, and neurological examinations were normal.

She had bicytopenia showing microcytic hypochromic RBC with nucleated RBC (nRBC) and hemoglobin of 4.6 g/dl with a reticulocyte index of 3.48; a total white cell count of 62 ×109/L and a platelet count of 60 ×109/L. Lactate dehydrogenase (LDH) was 2510 IU/L. Liver function test revealed direct hyperbilirubinemia (total bilirubin - 47.8mg/dL, direct bilirubin - 41 mg/dL). Kidney function revealed urea 103 mg/dl, creatinine 2.21 mg/dL. Prothrombin time and international normalized ratio (PT/INR) was 28s/ 2.15. C-reactive protein (CRP) was 192 mg/L. Serum procalcitonin was >75 ng/mL.

**Figure 1:**
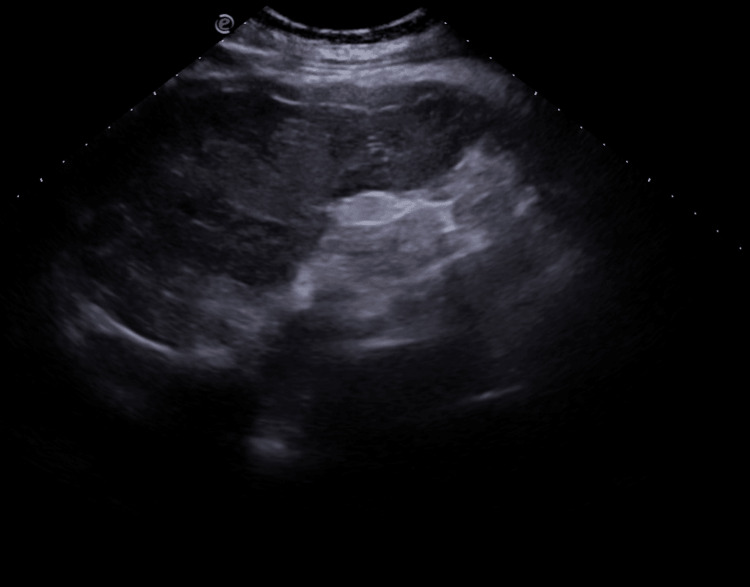
Ultrasonography of the abdomen Splenomegaly, with ill-defined confluent hypoechoic areas, is seen predominantly involving the peripheral portion of the spleen. The splenic capsule appears irregular, thickened, and echogenic with areas of subtle posterior acoustic shadowing suggestive of soft calcification. Associated hypoechoic areas are seen in the subcapsular region.

The patient was initially managed in line with sepsis with multiple organ dysfunction. In view of her high leukocyte count and procalcitonin, she was started on empirical antibiotics covering Community-acquired pneumonia and tropical infection. Since the patient had very high levels of conjugated bilirubin, an ultrasound abdomen was done. However, there were no features suggestive of biliary obstruction and cholangitis. A chest X-ray revealed left-sided pleural effusion. Arterial blood gases test (ABG) revealed moderate acute respiratory distress syndrome (ARDS), which improved with supplemental oxygen. Acute kidney injury improved after intravenous hydration. The patient had acute liver failure with grade 2 hepatic encephalopathy and deranged coagulation for which anti-hepatic coma measures were instituted with oral lactulose and rifaximin; however, no evidence of gastrointestinal bleeding was present. Bacterial and fungal blood cultures were negative. Bacterial culture of pleural fluid and urine cultures were negative. Dengue NS1 & IgM, malarial antigen by rapid immunochromatographic assay, scrub typhus IgM, leptospira IgM, hepatitis B and C, HIV serology, and autoimmune hepatitis panel were negative. She had worsening anemia and decreasing hematocrit despite multiple Packed RBC transfusions with high reticulocyte index; hemolytic anemia was suspected. Serum LDH was raised. Coombs' test was non-reactive. Ultrasound abdomen was suspicious of splenic sequestration (Figure 1)

**Figure 2:**
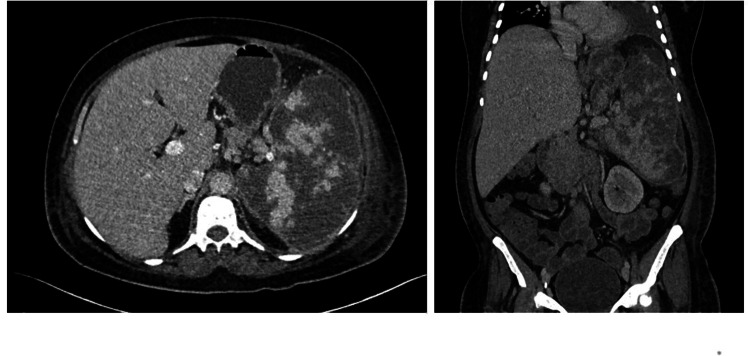
Post-contrast axial and sagittal images through the abdomen demonstrate an enlarged spleen and heterogeneous in density with numerous areas of low density which do not enhance, suggestive of splenic infarction

Contrast-enhanced computed tomography (CECT) thorax and abdomen was done to rule out the infectious sources, which revealed left-sided mild to moderate pleural effusion and splenomegaly with large areas of hypo/non-enhancing areas, suggestive of splenic sequestration (Figure 2)

High-performance liquid chromatography (HPLC) was done, and reports revealed hemoglobin S (Hb S) of 26.1 % (Table 1). The patient was diagnosed with sickle cell anemia with acute chest syndrome.

**Table 1:** High-performance liquid chromatography (HPLC) Hb - hemoglobin

Type of Hb	Percentage (%)
Hb A	56.1
Hb A2	3.1
Hb F	4.7
Hb S	26.1

Antibiotics were stopped. The patient received multiple packed RBC transfusions to maintain hemoglobin of more than 10 g/dL, and hematocrit >30% to prevent sickling and was started on hydroxyurea. Her bilirubin decreased gradually (total bilirubin - 3.3 mg/dL and direct bilirubin - 1.1 mg/dL) and sensorium improved. Left pleural effusion spontaneously resolved. Vaccination for *Haemophilus influenzae* type B, meningococcus, and pneumococcus was administered, and was discharged with close follow-up.

## Discussion

Sickle cell anemia is a chronic disorder with increasing global health importance, and India is estimated to have the second highest burden of disease in the world [3]. The presenting features of patients are related to hemolytic anemia and acute or chronic pain, and tissue ischemia because of vaso-occlusion. Acute chest syndrome is a major complication of sickle cell anemia which is responsible for higher ICU admission rates and mortality in these patients. Vaso-occlusion within pulmonary vasculature is the basis of ACS, which can be triggered by a variety of inciting events such as infection, asthma, infarction due to fat embolism, or venous thromboembolism. These events cause deoxygenation of Hb S and polymerization with subsequent sickling causing vaso-occlusion, ischemia, and endothelial injury [4]. ACS has a variable presentation in children and adults, with clinical courses being more severe in adults with an increased likelihood of mechanical ventilation and death. A majority of the ACS present with fever, chest pain, shortness of breath, and multi-lobe infiltrates in chest imaging, and almost 50 percent have pain preceding ACS [5].

A retrospective cohort study by Chaturvedi et al. described a distinct entity referred to rapidly progressive ACS, and multiorgan failure is recognized in up to one-fifth of adults with a history of ACS characterized by rapid progression of respiratory failure and often with multiorgan failure (acute kidney injury, hepatic dysfunction, altered mental status, multiorgan failure). He also found that declining platelet count at presentation was the only predictor of rapidly developing ACS [2]. In 2016 Chaturvedi et al. proposed diagnostic criteria for ACS stating that any new pulmonary density on chest imaging involving at least one complete lung segment and at least any one of the following, namely, temperature ≥38.5°C, >3 percent decrease in SpO2 (oxygen saturation) from a documented steady-state value on room air, tachypnea, intercostal retractions, nasal flaring, or use of accessory muscles of respiration, chest pain, cough, wheezing or rales [2]. A high level of suspicion is needed for a diagnosis of ACS since the presenting features can mimic vaso-occlusion crisis or multiorgan dysfunction due to sepsis. Pain control and respiratory support, either by non-invasive or invasive mechanical ventilation in case of severe respiratory failure, is often needed till the crisis tides over. Bronchodilators, fluid management, and empirical antibiotics in cases of suspected infection are needed. However, the mainstay of therapy for ACS is a simple exchange transfusion, which improves oxygenation and sickling, which halts the vicious cycle of acute chest syndrome. The goal of transfusion is to increase the hematocrit to 30 percent or the hemoglobin to 10-11 g/dL. Exchange transfusion can be done, which significantly lower Hb S levels. Usually, six to eight units of packed red cells are sufficient for full exchange transfusion in adults [6]. Concurrent management for preventing hypovolemia and venous thromboembolism prophylaxis should be done, which reduces prolonged hospital stays.

## Conclusions

Acute chest syndrome is a life-threatening complication of sickle cell crisis. It can masquerade as hemolytic jaundice and sepsis, requiring a high index of suspicion for diagnosis. Diagnosis is often delayed due to overlapping clinical and laboratory manifestations. Prompt diagnosis and timely intervention can prevent mortality and morbidity. In our case, the patient presented with clinical features compatible with obstructive jaundice, sepsis, and ARDS, which led to an initial investigation and treatment directed toward cholangitis and pneumonia. It is, therefore, essential to consider the strong possibility of underlying hematological disorder in the background of multiple blood transfusions. Clinicians managing suspected hemolytic anemia should have a high index of suspicion for diagnosing acute chest syndrome, which can mimic sepsis, respiratory distress, and multiple organ dysfunction, and can be managed by simple transfusion. This case highlights the uncommon presentation of acute chest syndrome masquerading as sepsis and obstructive jaundice, which can be challenging to diagnose. 
